# Endoplasmic reticulum-associated degradation mitigates atherosclerosis by maintaining cellular homeostasis

**DOI:** 10.3389/fphys.2025.1638694

**Published:** 2025-09-02

**Authors:** Haiming Niu, Lin Wu, Yingzhang Cai, Conghui Yu, Ning Lin, Xiaodong Cai, Miaolian Chen, Linli Wang

**Affiliations:** ^1^ Department of Critical Care Medicine, Zhongshan People’s Hospital, Zhongshan, China; ^2^ Department of Cardiology, The Third Affiliated Hospital of Sun Yat-sen University, Guangzhou, China; ^3^ Department of Traditional Chinese Medicine, Zhongshan People’s Hospital, Zhongshan, China; ^4^ Key laboratory of functional and clinical translational medicine, Xiamen Medical College, Xiamen, Fujian, China; ^5^ Zhongshan School of Medicine, Sun Yat-sen University, Guangzhou, China; ^6^ Bioland Laboratory, International Bioland, Guangzhou, China

**Keywords:** atherosclerosis, ERS, ERAD, Hrd1, vascular smooth muscle cell

## Abstract

Atherosclerosis (AS) is a fatal cardiovascular disease (CVD) that threatens human health. Although there are some treatments for AS in clinical practice, cardiovascular complications such as myocardial ischemia and hypoxia, heart failure, and stroke often occur in different AS subgroups. Therefore, it is critical and necessary to screen and identify novel protein molecules to mitigate this disease. Unstable plaques of AS is the main cause for fatal consequences, so it is particularly urgent to find a treatment to stabilize plaques to prevent cardiovascular and cerebrovascular diseases. During the formation of plaque, a large amount of protein is produced and misfolded; this process initiates endoplasmic reticulum stress (ERS). Despite unfolded protein response (UPR) in the clearing of unfolded proteins, endoplasmic reticulum (ER)-associated degradation (ERAD) maintains ER proteostasis in mammalian cells by degrading misfolded proteins. However, the role of ERAD has not been fully elucidated in AS. In this review, the role of ERS in the different cells that took part in AS was summarized; then, the rescue function of ERAD in all the cell types was elucidated, especially vascular smooth muscle cells. An updated summary of the recent studies and systematic knowledge of ERAD in the mechanism of AS was presented, which may help guide future research and provide novel insights into the prevention and treatment of related diseases.

## 1 Introduction

Atherosclerosis (AS) is the major pathological basis of cardiovascular disease, characterized by abnormal repair response of the vascular wall to chronic inflammation and metabolic imbalance (Song et al., 2020). In recent years, with the advancement of molecular biology technology, the molecular mechanism of AS has been gradually revealed, involving multidimensional networks such as lipid metabolism disorder (Weber and Noels, 2011), immunoinflammatory activation (Wolf and Ley, 2019), cell phenotypic transformation, and genetic regulation (Zheng et al., 2024). AS plaques originated from endothelial cells, smooth muscle cells, macrophages, and foam cells. Endothelial dysfunction is an early predictor of atherosclerosis, and the inflammatory response is accompanied with endothelial cell dysfunction (ECD), while high oxidative stress and reduced NO availability are the main causes of ECD, which increases vascular remodeling (Rui et al., 2021). Vascular smooth muscle cells (VSMCs) are highly specialized cells that play a principal role in atherosclerosis. As the major components of the vessel medium, VSMCs control vessel tone and diameter, both of which are key players in maintaining vascular tension and function. VSMCs’ proliferation, migration, and apoptosis can be induced by hypertension-initiated stretch stress and involve various signaling pathways, such as ERS and MAPK (Frismantiene et al., 2018). Stretch stress induced by hypertension could nonspecifically activate all transmembrane receptors of VSMCs, which initiates the process of AS^7^, but berberine could inhibit the adverse cellular behavior of VSMCs, thus influencing the progression of cardiovascular disease (CVD) (Wang L. et al., 2020). Stress in the endoplasmic reticulum (ER), a critical cellular organelle, a site of important biological processes, such as protein synthesis, folding, and modification (Schwarz and Blower, 2016), can be triggered by various pathological factors, and sustained or excessive ERS initiates the unfolded protein response (UPR), ultimately resulting in apoptosis and disease (Chen X. et al., 2023). The onset and advancement of AS involve ERS through different pathways, such as apoptosis, inflammatory response, oxidative stress, and autophagy (Mochida and Nakatogawa, 2022). However, there is another pathway that eliminates the unfolded proteins called endoplasmic reticulum-associated degradation (ERAD), which is hardly known in AS, so we provide a summary of how ERAD affects AS to explore the role of ERAD in atherosclerosis and its potential as a novel therapeutic target.

## 2 Molecular mechanism of endoplasmic reticulum stress (ERS) and endoplasmic reticulum-associated degradation

### 2.1 ER maintains cell homeostasis

**FIGURE 1:**
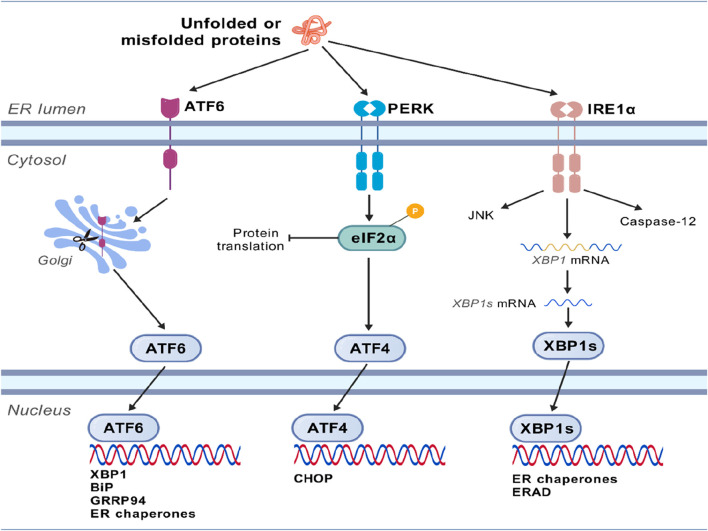
Molecular mechanism of endoplasmic reticulum stress. The unfolded or misfolded proteins accumulated in the ER initiate the unfolded protein response (UPR), which is primarily mediated by three transmembrane sensor proteins located on the ER membrane, namely, PERK, IRE1α, and ATF6. These three pathways work synergistically to enhance the protein-folding capacity of the endoplasmic reticulum, eliminate misfolded proteins, and reduce the input of new proteins. If endoplasmic reticulum stress persists and cannot be resolved, the UPR signal will shift from promoting survival to promoting apoptosis.

When unfolded or misfolded proteins accumulate in the ER, cells initiate the UPR to restore homeostasis. This response is primarily mediated by three transmembrane sensor proteins located on the ER membrane, namely, PERK, IRE1α, and ATF6.PERK (Oakes and Papa, 2015). Upon activation, eIF2α is phosphorylated to inhibit global protein translation to reduce the burden on the ER, while ATF4 is simultaneously activated, which then regulates genes involved in oxidative stress and amino acid metabolism and ultimately induces the expression of the pro-apoptotic factor CHOP. When IRE1α is activated, it exhibits both kinase and ribonuclease activities, which catalyze the non-canonical splicing of XBP1 mRNA, generating the active transcription factor XBP1s (spliced XBP1). XBP1s upregulates genes encoding ER chaperones and components of ER-associated degradation (ERAD). ATF6 is transported to the Golgi apparatus, where it is cleaved by proteases to release its cytoplasmic domain (ATF6f). ATF6f acts as a transcription factor, then translocates to the nucleus, and upregulates the expression of chaperone genes (such as BiP and GRP94) and XBP1 (Lebeaupin et al., 2018). These three pathways work synergistically to enhance the ER’s protein folding capacity, clear misfolded proteins, and reduce new protein influx. If ER stress persists and cannot be resolved, the UPR signaling switches from pro-survival to pro-apoptotic. This shift primarily occurs through CHOP expression, IRE1α-mediated activation of the JNK pathway, and caspase-12 (or its mammalian homologs) activation, ultimately inducing cell apoptosis (Merighi and Lossi, 2022), as shown in Figure 1.

**FIGURE 2:**
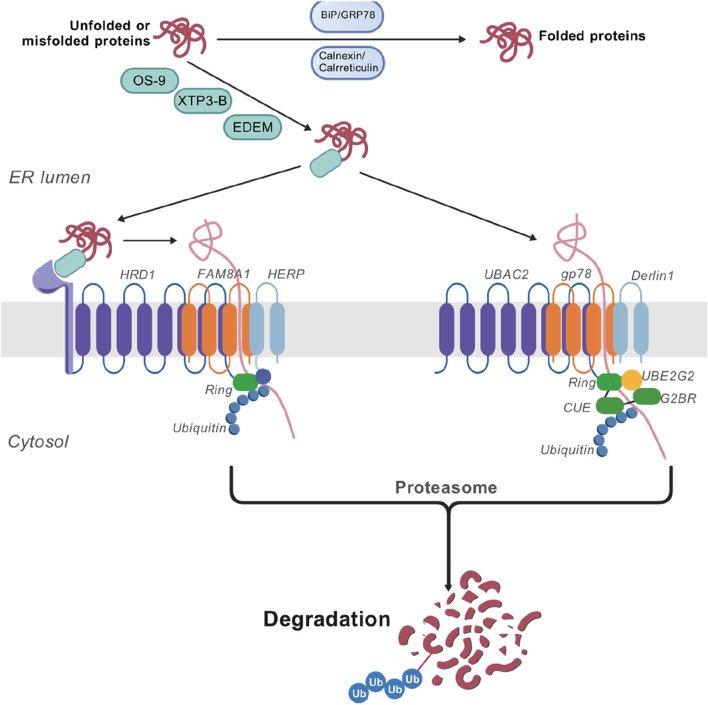
The mechanism of degradation of unfolded or misfolded proteins by the Hrd1 and gp78 complexes. The core mechanism of ERAD consists of two steps. First, molecular chaperones such as BiP/GRP78 and calnexin/calreticulin bind to unfolded proteins and attempt to promote their proper folding. If it fails, these misfolded proteins will be marked for degradation. The marked proteins will be recognized by the ubiquitin–ligase complex on the ER membrane (such as Hrd1 and gp78) and retrotranslocated into the cytosolic side of the ER membrane. Once exposed to the cytosolic face of the ER membrane, substrates are ubiquitinated and subsequently delivered to the proteasome for degradation.

ERAD is a quality control process for misfolded or unfolded proteins in the ER, which targets abnormal proteins for ubiquitination and delivery to proteasome degradation (Hwang and Qi, 2018). The core mechanism includes two steps. First, the process is initiated by recognition and targeting of molecular chaperones. The molecular mates such as BiP/GRP78 and calnexin/calreticulin bind to unfolded proteins and try to promote proper folding (Furmanik et al., 2021). If proper folding fails, the misfolded proteins are marked for degradation. The ER lectins (e.g., OS-9, XTP3-B, and EDEM) identify hydrophobic areas of abnormal glycosylation or exposure (Krshnan et al., 2022). Second, by activating the ubiquitination signal, transmembrane E3 ubiquitin ligase complexes (e.g., Hrd1 and gp78) recognize substrates and initiate ubiquitination (Krshnan et al., 2022; Li et al., 2021; Sun and Brodsky, 2019; Wei et al., 2024). This process is essential for removing potentially harmful protein aggregates and maintaining cell function (Bhattacharya and Qi, 2019); the following figure shows the relationship between ERAD and ERS. ERAD has several branches (ubiquitin ligases), some of which are similar between yeast and mammalian cells. One branch is HRD1, also known as synoviolin (SYVN1), contains a unique membrane subunit, FAM8A1, which facilitates the interaction between HERP and HRD1 (Schulz et al., 2017). Reports also provided evidence for remodeling of HRD1 complexes on ERS (Eura et al., 2020). Consistent with its importance in ERAD, the HRD1 complex has several substrates, many of which have disease relevance (Bhattacharya and Qi, 2019; Qi et al., 2017). Critical residues in Hrd1 were responsible for the degradation of membrane substrates but dispensable for luminal substrates, which were identified by mutagenesis studies (Sato et al., 2009). Another ubiquitin ligase is gp78, also known as AMFR, which has a distinct set of membrane partners, mainly binding to Derlin-1 and UBAC2. In its carboxy-terminal tail, gp78 has a RING domain, a ubiquitin-binding CUE domain, and a G2BR motif involved in high-affinity binding to the UBE2G2 conjugating enzyme (Das et al., 2009). These gp78 cytosolic domains stimulate processive assembly of ubiquitin chains and their transfer to substrates (Li et al., 2007). Another is TEB4, also known as MARCHF6 (membrane-associated RING C3HC4), has an amino-terminal RING domain, followed by a large membrane domain containing 14 TMDs and carboxy-terminal elements (CTE) (Zattas and Hochstrasser, 2015). Similar to Doa10, TEB4 targets the membrane and soluble substrates in the cytosol. In contrast, there are no reports evidencing the role of TEB4 in the degradation of nuclear proteins. Other ubiquitin ligases, such as RNF139/TRC8, RNF145, and RNF5, need no further elaboration. The ER-membrane ubiquitin–ligase complex capable of recognizing substrates in the lumen or membrane of the ER defines each ERAD branch and promotes their transfer into the cytosol for ubiquitination. The core mechanism of ERAD includes the following steps. The first step is recognition and tagging of misfolded proteins. ER-resident chaperones (e.g., BiP/GRP78 and calnexin/calreticulin) bind to hydrophobic regions of unfolded proteins to identify misfolded or unassembled proteins (Kırça and Yeşilkaya, 2021). EDEM/OS-9 family proteins are further screened and targeted to the ERAD pathway (Zhou et al., 2020; Frachon et al., 2024). The second step is ubiquitination and retrotranslocation; in E3 ubiquitin ligase-mediated ubiquitination, the key E3 ligases (e.g., Hrd1, gp78, and CHIP) attach ubiquitin chains to target proteins, marking them for proteasomal recognition. Target proteins are translocated from the ER lumen to the cytoplasm via the Sec61 channel or the Derlin-1 complex (Needham et al., 2019). The third step is proteasomal degradation; ubiquitinated proteins are extracted to the cytoplasm via the p97/VCP (valosin-containing protein) complex and ultimately degraded by the 26S proteasome (Weihl et al., 2006). The molecular mechanism is shown in Figure 2.

## 3 Role of endoplasmic reticulum stress in AS

### 3.1 ERS plays an important role in the occurrence and development of AS

The effects are mainly exerted by altering the functions of endothelial cells, macrophages, and smooth muscle cells, thereby promoting inflammation, lipid metabolism disorders, and apoptosis (Zhang et al., 2023), which function in the following aspects on AS. ERS induces endothelial cell dysfunction through increased inflammation and upregulation of adhesion molecules such as VCAM-1 and ICAM-113, while also promoting endothelial release of inflammatory factors (e.g., IL-6) via IRE1α and PERK pathways, thereby intensifying monocyte adhesion and migration to the vascular intima (Yingchun et al., 2018). In another way, ERS interacts with oxidative stress, resulting in decreased nitric oxide (NO) bioavailability of endothelial cells and aggravating vasodilation dysfunction (Lenna et al., 2014). ERS affects macrophage/foam cell formation in lipid accumulation and apoptosis (Cui et al., 2023), which is activated after macrophage phagocytosis of oxidized low-density lipoprotein (oxLDL), interfering with cholesterol reversal transport (such as inhibiting ABCA1 expression) and promoting foam cell formation (Xue et al., 2022). Persistent ERS leads to macrophage death through the CHOP-mediated apoptotic pathway, releasing lipids to form plaque necrotic cores (Sukhorukov et al., 2020). Next, amplification of inflammation, such as activation of the NF-κB pathway, promotes the secretion of TNF-α, IL-1β, and other inflammatory factors and accelerates plaque progression (Cao et al., 2016). Moreover, ERS affects phenotypic transformation of smooth muscle cell (SMC) apoptosis and plaque instability. ERS induces SMC apoptosis (via CHOP and JNK pathways) (Wang L. et al., 2020), leading to thinning of the fiber cap and susceptibility to plaque rupture, finally causing imbalance of migration and proliferation, even vascular occlusion. ERS may also promote SMC migration to intima and excessive proliferation, increase plaque volume, and exacerbate vascular wall fibrosis (Wang L. et al., 2023). On the other hand, ERS interferes with liver lipoprotein synthesis and peripheral tissue lipid processing and increases circulating LDL levels. Finally, by activating the systemic inflammatory response, ERS then leads to a chronic inflammatory environment and accelerates AS progression by releasing DAMP-related molecular patterns (DAMPs) (Bian et al., 2022). ERS may regulate lipid metabolism by restoring ABCA1 expression or enhancing autophagy, promoting cholesterol outflow, and reducing foam cell formation. ERS inhibitors, such as the chemical chaperone 4-PBA and TUDCA, may alleviate protein misfolding and improve cell function. However, pathway-specific interventions such as targeting IRE1α, PERK, or ATF6 signaling (such as small-molecule inhibitors) may reduce inflammation and apoptosis (Salvagno et al., 2022). Intervention strategies targeting the ERS pathway may provide new directions for AS treatment, but their two-sided effects (protective stress vs pathological injury) need further studies to optimize target selection.

## 4 Endoplasmic reticulum-associated degradation in different cell types of AS endothelial cell parts

Endothelial cell dysfunction plays a key role in the occurrence and progression of AS, and its mechanisms involve multiple interrelated pathological processes (Gimbrone and García-Cardeña, 2016). Endothelial injury leads to the breakdown of tight connections, allowing macromolecules such as LDL to penetrate into the vascular intima, which are deposited and oxidized (ox-LDL) to form lipid streaks (Bai et al., 2020). Subcutaneous matrix modification promotes lipid retention and initiates plaque formation, resulting in upregulated expression of adhesion molecules such as VCAM-1 and ICAM-1, which promotes the adhesion of monocytes and T cells and then migration to the endodermis (Yang et al., 2024; Wang X. et al., 2023). MCP-1 recruits monocytes and differentiates into macrophage and phagocyte ox-LDLs, forming foam cells and constituting the plaque lipid core (Vinchi et al., 2020). The cytokines in chronic inflammatory cycle, such as IL-6 and TNF-α, are continuously released to maintain the inflammatory microenvironment (Kwaifa et al., 2020). Coagulation–anticoagulation imbalance and procoagulant phenotypic transition are reflected in the release of tissue factor (TF), activating the exogenous clotting pathway, and vWF promotes platelet aggregation and increases the risk of thrombosis (Manz et al., 2022; Ma et al., 2015). The reduced synthesis of anticoagulant substances like nitric oxide (NO) and prostacyclin (PGI_2_) weakens antiplatelet and vasodilation function, while decreased endothelial nitric oxide synthase (eNOS) activity leads to vasoconstriction (e.g., increased endothelin-1), elevates blood pressure, and increases oxidative stress (Zhao et al., 2020). Interestingly, the secretion of PDGF and other factors promotes the migration of smooth muscle cells into the intima and proliferation to form fiber caps (Li et al., 2018). Finally, ROS-accelerated apoptosis of ECs in the late stage leads to plaque instability (Evrard et al., 2016). The main role of ERAD is to maintain endoplasmic reticulum homeostasis, protein quality control, and error-protein clearance (Qi et al., 2017). ERAD ensures the correctness of endothelial secreted proteins (e.g., vWF and clotting factors) by recognizing, reverse transporting, ubiquitinating, and degrading misfolded or unassembled proteins (e.g., vasoactive and adhesion molecules). Normal ERAD activity is the basis for vascular permeability, coagulation regulation, and inflammatory response (Ji et al., 2023). Under conditions such as hypoxia, oxidative stress, or hyperglycemia, ERAD is activated to clear accumulated abnormal proteins and prevent apoptosis resulting from overactivation of the UPR (Hwang and Qi, 2018). Moderate ERAD promotes cell adaptation to stress, while functional deficiencies may lead to chronic ERS, triggering endothelial cell apoptosis (such as atherosclerotic plaque formation) (Chapple et al., 2013). Regulation of vascular function is associated with angiogenesis and repair, indicating that ERAD regulates the stability of signaling proteins such as VEGF receptors and affects angiogenesis (such as abnormal angiogenesis in diabetic retinopathy) (Chen et al., 2015). By degrading inflammation-related proteins such as cytokine receptors, ERAD may inhibit the proinflammatory response of endothelial cells (Chen S. et al., 2023). oxLDL inhibits ERAD activity, exacerbating ERS and endothelial damage (Hong et al., 2014). Moreover, hyperglycemia leads to ERAD overload, promoting increased vascular permeability and dysfunction (endoplasmic reticulum-associated degradation, 2019). Key molecules and pathways of E3 ubiquitin ligases (e.g., Hrd1 and gp78) may specifically label faulty proteins in endothelial cells and influence disease progression (Li et al., 2020). Sel1L–Hrd1 complexes may have unique substrate selectivity in endothelial cells (Wei et al., 2024; Lin et al., 2024) and recognize transmembrane protein. Moreover, targeting ERAD regulation may have therapeutic potential; for example, enhancing ERAD efficiency or alleviating ERS may be strategies for treating endothelial dysfunction (e.g., using chaperon-assisted protein folding or inhibiting excessive UPR signaling) (Christianson et al., 2023). Future studies need to further reveal the specific regulatory network of ERAD in endothelial cells to develop precise treatment strategies. The key findings of ERAD in cardiovascular diseases are summarized in Table 1.

**TABLE 1:** Summary of the key findings of ERAD in cardiovascular diseases.

Disease	Cell	Target	Reference
AS	VSMC	Hrd1	89
AS	Macrophages	ERAD	95
Bartter syndrome	Renal cells	AUP1	84
Obesity	Brown adipocytes	ERAD	83

### 4.1 Macrophage parts

Macrophages play central roles in atherosclerosis pathogenesis, in which monocytes differentiate into macrophages upon endothelial injury, internalizing oxidized LDL via scavenger receptors (e.g., CD36) to form foam cells that establish plaque lipid cores (Barrett, 2020; Blagov et al., 2023). They amplify inflammation by secreting cytokines (TNF-α and IL-1β) and MMPs, destabilizing plaques via collagen degradation, while their polarization dictates outcomes: pro-inflammatory M1 macrophages exacerbate plaque vulnerability, whereas M2 macrophages promote stability through efferocytosis and anti-inflammatory mediators (IL-10 and TGF-β) (Yu et al., 2023; Hou et al., 2023). Chronic lipid overload triggers macrophage death, releasing necrotic debris that enlarges plaque necrotic cores. Therapeutic strategies targeting lipid uptake, NLRP3 inflammasome inhibition, or efferocytosis enhancement may mitigate disease progression (Orecchioni et al., 2022). The ERAD pathway plays a central role in macrophages by maintaining proteostasis and regulating inflammatory signaling networks. Upon immune activation or pathogen infection, ERAD collaborates with the UPR to eliminate misfolded proteins (e.g., excess cytokines or pathogen-derived proteins), thereby preventing excessive ERS-induced apoptosis. Concurrently, ERAD finely modulates NF-κB/MAPK signaling pathways through the degradation of specific regulatory molecules (e.g., TRAF2 or NLRP3 inflammasome components), balancing the secretion of pro-inflammatory cytokines (such as TNF-α and IL-6) and optimizing MHC-I antigen presentation to enhance adaptive immunity. In anti-infection defense, ERAD directly targets viral proteins (e.g., influenza hemagglutinin) or counteracts pathogen immune evasion strategies (e.g., herpesvirus US11-mediated MHC-I degradation) (Huang et al., 2024). However, ERAD dysfunction disrupts macrophage homeostasis, exacerbating atherosclerotic plaque necrosis, autoimmune diseases driven by self-antigen accumulation, and obesity-associated metabolic inflammation. These roles highlight ERAD as a critical node in the immunometabolism network and a potential therapeutic target for related pathologies. However, further research needs to elucidate the mechanism between ERAD and macrophage polarization.

### 4.2 Vascular smooth muscle cell parts

The regulation of vascular tone and blood flow relies largely on proper VSMC activity and physiological functioning of the vasculature (Lechartier et al., 2022). VSMCs are differentiated, contractile, stationary, and quiescent in the healthy vasculature (Bennett et al., 2016). In addition, the contractile proteins include smooth muscle α-actin (SM-α-actin), SM myosin heavy chain, calponin, SM 22α, smoothelin, and SM myosin light chain (MLC) (Touyz et al., 2018). Meanwhile, under some conditions, VSMCs may shift from the contractile phenotypes to synthetic phenotypes (Elmarasi et al., 2024). Importantly, the VSMC phenotype switch is necessary during development and vascular remodeling for the formation and maturation of vessels (Lambert and Jørgensen, 2025), while this phenotype switch may be dysregulated in atherosclerosis, aortic aneurysm, or hypertension (Petsophonsakul et al., 2019). VSMCs that undergo phenotypic transformation involve MAPK signaling (Wang X. et al., 2020), epigenetic changes (Lambert and Jørgensen, 2025), and endoplasmic reticulum stress (Ren et al., 2021; Pan et al., 2024; Owens et al., 2004). During vascular development, SMC shows high plasticity, characterized by high rates of proliferation, migration, and production of extracellular matrix components such as collagen, elastin, and proteoglycans, which constitute a major portion of the blood vessel wall, while simultaneously acquiring contractile capabilities (Chen R. et al., 2023; Cao et al., 2022). Secretory and transmembrane protein folding and translocation occur in the ER, which regulates cellular Ca^2+^ uptake, storage, and signaling, and participates in the production of cellular lipids, such as cholesterol, glycerophospholipids, and ceramides (Ren et al., 2021). ERS regulates cell survival, apoptosis, and phenotypic switching in VSMCs through the UPR, exerting dual effects on vascular homeostasis and disease progression (Ivanova and Orekhov, 2016). Under pathological conditions (e.g., hypertension and atherosclerosis), chronic ERS activates PERK/ATF4, IRE1α/XBP1, and ATF6 pathways, inducing oxidative stress, inflammatory cytokine secretion, and calcium signaling dysregulation. This promotes VSMC transition from a contractile to a synthetic phenotype, enhancing migration and proliferation, thereby driving vascular remodeling (Clément et al., 2019). Excessive ERS also triggers CHOP-mediated apoptosis, exacerbating plaque instability and vascular dysfunction (Tóth et al., 2024). Moderate ERS, however, helps maintain cellular function by clearing misfolded proteins, suggesting that targeted regulation of ERS may offer a potential therapeutic strategy for vascular diseases. The lipid accumulation in VSMCs has been linked to the upregulation of sterol regulatory element-binding protein 2 (SREBP-2) (Ma et al., 2013), and treatment with homocysteine has also been shown to activate ERS. Diabetes mellitus, frequently associated with atherosclerosis, may involve glucosamine accumulation in vascular cells, which can induce ERS accompanied by GRP78 upregulation (Furmanik et al., 2021). Further studies are needed to elucidate the role of ERS signaling in smooth muscle cells during the development of atherosclerosis (Chengji and Xianjin, 2018). In VSMCs, ERAD not only maintains proteostasis but is also closely associated with pathological processes such as atherosclerosis, vascular calcification, and vascular remodeling. These functions mainly include the core molecular mechanisms of ERAD. Pathology-specific regulation of ERAD in VSMCs is modulated by pathological factors (e.g., oxidative stress, inflammatory cytokines, and lipotoxicity) and interacts with key pathological processes (Liu et al., 2005).

VSMC phenotypic switching and proliferation/apoptosis regulation are mediated by ERAD during atherosclerosis (Grootaert and Bennett, 2021). The increase in protein synthesis triggered ERS and ERAD activation (Wang L. et al., 2023). This process involves reactive oxygen species, and HRD1 deficiency may magnify the phenotypic change (Wang et al., 2024a); moreover, HRD1 alleviates VSMC senescence by inhibiting ERS-induced ROS production (Wang et al., 2024b). These results demonstrate ERAD functions through the life cycle of VSMCs. Wang et al. found that cholesterol induces the deficiency of HRD1 in VSMCs, along with mitochondria dysfunction, indicating the interaction between the ER and mitochondria (Wang et al., 2024b). Another mechanism is pro-survival vs pro-apoptotic roles. ERAD alleviates ERS by clearing misfolded proteins, promoting VSMC survival (e.g., via Hrd1-dependent degradation). An apoptotic trigger such as ERAD failure (e.g., E3 ligase deficiency) leads to unfolded protein accumulation, activating the CHOP pathway to induce VSMC apoptosis and necrotic core formation in plaques (Chen et al., 2021). Chronic ERS activates the ATF4/XBP1 pathway to promote VSMC osteogenic differentiation, which is exacerbated by ERAD deficiency (Zhu et al., 2024). Inflammation and extracellular matrix (ECM) remodeling, driven by inflammatory cytokine release, are exacerbated by ERAD deficiency, which activates the IRE1α-XBP1 pathway and increases the secretion of IL-6 and TNF-α, thereby promoting vascular inflammation. ERAD influences ECM (Koloko Ngassie et al., 2024) stability by degrading abnormal collagen and elastin, impacting fibrous cap integrity in plaques. ERAD affects VSMC pathophysiology in AS plaque stability: ERAD regulates VSMC survival–apoptosis balance, affecting fibrous cap thickness. Ox-LDL induces ERS in VSMCs, and impaired ERAD activity promotes lipid accumulation and foam cell formation (Stevenson et al., 2016). Targeting HRD1 may block the phenotypic transition from contractile to synthetic, deeply inhibiting the senescence process and preventing VSMC-initiated diseases. However, more animal studies are still needed to verify the hypothesis.

### 4.3 ERAD affects all stages of AS

**FIGURE 3:**
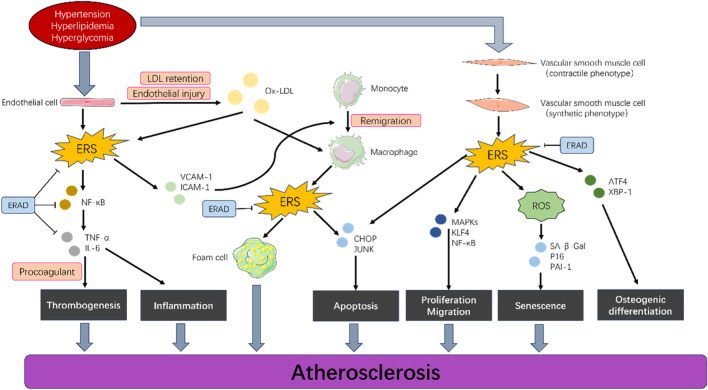
Endoplasmic reticulum-associated degradation in different cell types of AS. Hypertension, hyperlipidemia, and hyperglycemia induce proliferation, migration, apoptosis, and senescence in vascular endothelial cells, smooth muscle cells, and macrophages by activating a series of signaling pathways (such as endoplasmic reticulum stress) in these cells, which ultimately lead to the development of AS. ERAD can effectively control this progression.

Pathologically, AS is divided into four stages: the fatty streak stage, in which ERAD promotes the progression of AS by affecting endothelial cells and macrophages; the fibrous plaque stage, atheromatous plaque stage, and complicated lesion stage. However, current research does not have clear data on the role of ERAD in each stage. However, there are few data to report the effects of ERAD and the influence of these cells in AS. Studies have shown that plasma GRP78/BiP concentrations are elevated in patients with metabolic disorders and subclinical atherosclerosis (Girona et al., 2019). GRP78/BiP may be a useful marker for metabolic and cardiovascular risks. In addition, platelets can induce ERS and UPR in macrophages through thermosensitive membrane proteins (Derler et al., 2024), and this process is independent of the inflammatory activation of macrophages. Aging and an atherogenic diet activate different UPR pathways, leading to different vascular responses. Compared with dietary intervention, aging is associated with impaired ER protein folding function and increased aortic cell apoptosis (Zhou et al., 2021). However, currently no clear data on detailed studies of each stage of AS exist. This review can provide ideas for subsequent detailed studies on the mechanism of ERAD in AS. The molecular mechanism of ERAD in AS is shown in Figure 3.

## 5 Therapeutic potential of ERAD modulators for treatment of atherosclerosis

### 5.1 HRD1 modulates VSMC

Using high-fat diet (HFD)-fed mice and *in vitro* models, researchers found that HRD1 expression was significantly reduced in aortic tissues under cholesterol-rich conditions, correlating with VSMC dedifferentiation (loss of contractile markers like SMMHC and α-SMA) and increased ERS, inflammation, oxidative stress, and extracellular matrix remodeling. Cholesterol exposure in VSMCs exacerbated HRD1 downregulation, ERS, and activation of pro-proliferative/migratory pathways (e.g., KLF4, ROS, and JNK/NF-κB). CRISPR-mediated HRD1 knockout in VSMCs amplified these effects, enhancing proliferation, migration, and ERS, while ERS inhibition (via 4-PBA) mitigated these changes. Transcriptomic analysis revealed that HRD1 deficiency upregulated expressions of genes linked to ERS, proliferation, and migration while suppressing those of contractile markers (Wang L. et al., 2023). The study highlights HRD1 as a critical regulator of VSMC contractile homeostasis and suggests its therapeutic potential in combating metabolic disorder-driven vascular remodeling. In human atherosclerotic plaques and high-fat diet-fed ApoE^−/−^ mice, HRD1 expression was significantly decreased, correlating with increased ERS markers (BiP and IRE1α), senescence markers (SA-β-Gal, P16, and PAI-1), and mitochondrial dysfunction. Cholesterol exposure or CRISPR-mediated HRD1 knockout in VSMCs amplified senescence, inflammation, and mitochondrial ROS, while ERS inhibition (4-PBA) or ROS scavengers (NAC and MitoTEMPO) partially reversed these effects. HRD1 overexpression mitigated cholesterol-induced ERS, ROS, and senescence, restoring VSMC homeostasis. These findings identify HRD1 as a critical regulator of VSMC senescence and a potential therapeutic target for vascular aging-related diseases (Wang et al., 2024b). However, precise animal studies relating to ERAD in vascular function need to fill the gap in this area.

Targeting the ERAD pathway offers a novel therapeutic strategy for atherosclerosis. ERAD maintains ER homeostasis by identifying and clearing misfolded proteins, and its dysfunction is closely linked to ERS in macrophages during atherosclerosis. Enhancing ERAD activity (e.g., via small-molecule activators or genetic modulation) can alleviate ERS, reduce excessive activation of the UPR, and inhibit pro-inflammatory cytokine release (e.g., IL-6 and TNF-α) (Xu and Fang, 2020) and apoptosis signaling pathways (e.g., CHOP and caspase-12), thereby protecting plaque macrophages, delaying foam cell formation, and stabilizing necrotic cores. Additionally, ERAD synergizes with the proteasome to degrade oxidatively modified proteins, potentially mitigating oxLDL-induced lipotoxicity and improving plaque stability.

### 5.2 ERAD demonstrates promising clinical potential in the treatment of atherosclerosis

The core rationale lies in the fact that key cells within atherosclerotic lesions (such as macrophages, endothelial cells, and smooth muscle cells) frequently experience ERS (Ren et al., 2021). As the central mechanism for clearing misfolded proteins and alleviating ERS, ERAD would become dysfunctional, exacerbating the pro-inflammatory and pro-apoptotic signals of the UPR (Travers et al., 2000). This dysfunction promotes lipid accumulation, inflammation, foam cell formation, and plaque instability. Consequently, therapeutic targeting of the enhancement or modulation of the ERAD pathway (e.g., via small-molecule drugs activating key ERAD components like E3 ubiquitin ligases, deubiquitinating enzymes, or co-factors) emerges as a potential strategy. This approach holds promise for alleviating ERS by more efficiently clearing misfolded proteins and toxic metabolites (e.g., oxidized lipid-modified proteins); it mitigates cellular damage caused by excessive UPR activation. Another approach is inhibiting the inflammatory response by reducing the release of ERS-driven inflammatory cytokines (such as IL-6 and TNF-α) and inflammasome activation, thereby lowering intra-plaque inflammation. The third is reducing cellular apoptosis by protecting endothelial cells and smooth muscle cells, enhancing plaque stability. The fourth is improving lipid metabolism by indirectly regulating lipid homeostasis, potentially by influencing the stability of key proteins involved in cholesterol reverse transport or lipoprotein metabolism, thereby reducing lipid accumulation and foam cell formation within macrophages (Zelcer et al., 2014). The fifth is stabilizing vulnerable plaques by comprehensively reducing inflammation, decreasing apoptosis and the necrotic core, and promoting fibrous cap integrity, which lowers the risk of plaque rupture and thrombosis (Kong et al., 2016). Although still in the research stage and facing challenges such as target specificity and delivery methods, strategically enhancing or restoring ERAD function through small-molecule drugs or biologic agents offers an innovative therapeutic approach for atherosclerosis based on proteostasis regulation. This approach could potentially synergize with existing lipid-lowering and anti-inflammatory therapies in the future.

Selective inhibition of ERAD-associated factors (e.g., the E3 ubiquitin ligase HRD1 or the OST complex) may modulate specific pathological processes. For instance, in advanced plaques, overactivated ERAD may exacerbate inflammation by degrading anti-inflammatory or repair proteins (e.g., IL-10 receptor), and targeted suppression of specific ERAD components could restore protective signaling (Liu et al., 2021). Furthermore, the interplay between ERAD and autophagy (e.g., via p62/SQSTM1) highlights opportunities for combination therapies, such as pairing ERAD activators with autophagy inducers to synergistically clear toxic protein aggregates (Wu et al., 2025). However, ERAD modulation requires spatiotemporal precision to avoid disrupting normal protein quality control, and clinical translation faces challenges in targeted delivery and cell-type specificity.

The strategies include cell-specific delivery using nanocarriers to target VSMCs, minimizing systemic toxicity. Therapeutic strategies targeting ERAD, such as the development of small-molecule inhibitors or enhancers, show potential for the treatment of cardiovascular diseases. However, these strategies need to be precisely regulated to avoid potential side effects.

Another future research should focus on understanding the specific role of ERAD in different cardiovascular diseases and how this knowledge can be used to develop new treatments. New technologies, such as high-throughput screening and gene editing, will help reveal the complexity of ERAD and its potential therapeutic value in cardiovascular disease.

## 6 Conclusion

The role of ERAD in cardiovascular diseases is an emerging research area with important clinical significance. Further studies will help uncover the specific mechanisms of ERAD in cardiovascular pathophysiology and may lead to the development of new treatment strategies.
